# Cancer and psychiatric diagnoses in the year preceding suicide

**DOI:** 10.1002/cam4.5201

**Published:** 2022-09-17

**Authors:** Geoffrey D. Kahn, Samantha H. Tam, Julia W. Felton, Joslyn Westphal, Gregory E. Simon, Ashli A. Owen‐Smith, Rebecca C. Rossom, Arne L. Beck, Frances L. Lynch, Yihe G. Daida, Christine Y. Lu, Stephen Waring, Cathrine B. Frank, Esther O. Akinyemi, Brian K. Ahmedani

**Affiliations:** ^1^ Center for Health Policy & Health Services Research Henry Ford Health Detroit Michigan USA; ^2^ Department of Otolaryngology – Head and Neck Surgery Henry Ford Health Detroit Michigan USA; ^3^ Kaiser Permanente Washington Health Research Institute Seattle WA USA; ^4^ Department of Health Policy and Behavioral Sciences Georgia State University School of Public Health Atlanta Georgia USA; ^5^ HealthPartners Institute Minneapolis Minnesota USA; ^6^ Institute for Health Research, Kaiser Permanente Colorado Aurora Colorado USA; ^7^ Center for Health Research Kaiser Permanente Hawaii Portland Oregon USA; ^8^ Center for Integrated Health Care Research Kaiser Permanente Hawaii Honolulu Hawaii USA; ^9^ Department of Population Medicine Harvard Medical School and Harvard Pilgrim Health Care Institute Boston Massachusetts USA; ^10^ Essentia Institute of Rural Health Duluth Minnesota USA; ^11^ Department of Psychiatry Henry Ford Health Detroit Michigan USA

**Keywords:** behavioral science, cancer management, epidemiology, quality of life

## Abstract

**Background:**

Patients with cancer are known to be at increased risk for suicide but little is known about the interaction between cancer and psychiatric diagnoses, another well‐documented risk factor.

**Methods:**

Electronic medical records from nine healthcare systems participating in the Mental Health Research Network were aggregated to form a retrospective case–control study, with ICD‐9 codes used to identify diagnoses in the 1 year prior to death by suicide for cases (*N* = 3330) or matching index date for controls (*N* = 297,034). Conditional logistic regression was used to assess differences in cancer and psychiatric diagnoses between cases and controls, controlling for sex and age.

**Results:**

Among patients without concurrent psychiatric diagnoses, cancer at disease sites with lower average 5‐year survival rates were associated with significantly greater relative risk, while cancer disease sites with survival rates of >70% conferred no increased risk. Patients with most psychiatric diagnoses were at higher risk, however, there was no additional risk conferred to these patients by a concurrent cancer diagnosis.

**Conclusion:**

We found no evidence of a synergistic effect between cancer and psychiatric diagnoses. However, cancer patients with a concurrent psychiatric illness remain at the highest relative risk for suicide, regardless of cancer disease site, due to strong independent associations between psychiatric diagnoses and suicide. For patients without a concurrent psychiatric illness, cancer disease sites associated with worse prognoses appeared to confer greater suicide risk.

## Graphical abstract

Relative log‐odds of suicide among patients with concurrent cancer and psychiatric disorder diagnoses.

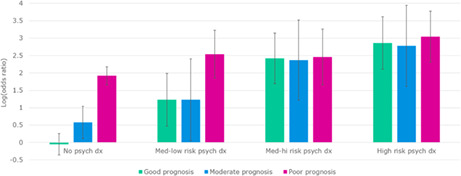

Suicide is one of the top ten leading causes of death in the US and the steady increase in suicide mortality rates across nearly all sectors of the population has become a nationally recognized public health crisis.^1^, ^2^ A variety of physical and psychiatric illnesses have been shown to be associated with suicide, including cancer, COPD, HIV/AIDS, depression, anxiety, and personality disorders.^3^, ^4^, ^5^, ^6^, ^7^, ^8^, ^9^ Across physical illnesses, the evidence for an increased suicide risk among people diagnosed with cancer is particularly robust. A recent systematic review and meta‐analysis identified 22 studies on the subject and concluded that cancer patients had a 55% greater risk of suicide than the general population (pooled standardized mortality ratio 1.55, 95% CI 1.37–1.74).^4^ The relative risk varied by cancer site, from a low of 1.24 (1.03–1.48) in breast cancer to a high of 3.07 (2.20–4.28) in lung, bronchus, and trachea cancers, with melanoma and skin cancers showing no increased risk (0.93, 0.75–1.16).^4^ Overall, there is a robust trend showing that cancers with worse prognoses tend to be associated with higher relative risk of suicide. The authors note that research is needed on the role of comorbid psychiatric disorders and cancer.

Despite considerable evidence suggesting their unique contributions to suicide risk, few studies have examined the interaction between psychiatric disorders and cancer diagnoses on suicide risk. A large, multisite study of US healthcare systems concluded that past‐year cancer diagnosis was associated with a 40% greater relative odds of suicide (OR 1.40, 1.21–1.62) even when controlling for past‐year psychiatric diagnosis and demographic characteristics.^3^ However, this study did not assess whether the relative risk was different between individuals with and without a concurrent psychiatric diagnosis. Identifying which cancer patients are at the highest suicide risk can help ensure the highest risk patients receive supportive oncology care that includes suicide risk mitigation interventions, or at least risk screening, as appropriate for each individual patient. Over the next several decades, as the US population ages and the entirety of the Baby Boom cohort surpasses age 65, the incidence of cancer is projected to increase by nearly 50%, from 1,534,500 in 2015 to 2,286,300 in 2050.^10^ Additionally, the 2020 National Survey on Drug Use and Health (NSDUH) suggested that 21% of the US population is living with a psychiatric disorder.^11^ These numbers suggest that understanding how psychiatric disorders and cancer diagnoses interact to impact suicide risk is important and relevant to a large segment of the US population.

In the current study, we expand on prior work examining the association between cancer and suicide^3^ and psychiatric disorders and suicide^12^ by parsing patients by cancer site (as a surrogate for prognosis) and specific psychiatric diagnosis, and examining possible interactive effects between these factors. The aim was to identify any synergistic effects between these known risk factors to better enable healthcare workers to stratify patients and respond appropriately based on their suicide risk. This work aligns with national priorities around suicide prevention. In 2021, the Surgeon General's Call to Action to Implement the National Strategy for Suicide Prevention was the latest update to a series of reports providing evidence‐informed guidelines and aspirational goals and objectives for reducing suicide mortality and morbidity.^13^ Action 4.2 specifically calls on providers to, “Improve suicide risk identification in health care settings.” By leveraging 15 years of data from a consortium of healthcare systems across the US, we were able to examine the association between suicide mortality and cancer and mental health diagnoses with a high level of granularity, allowing us to identify more specific sets of patient characteristics that may be indicative of elevated suicide risk and thus warrant targeted preventive intervention from the healthcare system.

## 1 METHODS

### 1.1 Study sample

Data came from a subset of healthcare systems participating in the Mental Health Research Network (MHRN), a consortium of learning healthcare systems across 15 states (“Mental Health Research Network [MHRN],”^14^, ^15^). Specifically, nine systems contributed data from their electronic medical records: Essentia Institute of Rural Health (Minnesota, Wisconsin, and North Dakota), HealthPartners (Minnesota, Wisconsin), Harvard Pilgrim Healthcare (Massachusetts), Henry Ford Health System (Michigan), and Kaiser Permanente health systems in Colorado, Georgia, Hawaii, Oregon, and Washington. Data from these sites were pooled to form a retrospective case–control study.

Cases (*N* = 3330) were defined as individuals who died by suicide between 2000 and 2015, were members of the participating health systems, and were continuously enrolled in their health plan for ≥10 months in the year prior to death. This last criterion was used to capture all healthcare utilization occurring before suicide while allowing a short gap given that individuals are often disenrolled from health plans in the month of their death. Controls were a random selection of 100 members per case enrolled at the same health system during the same year in which the case died. The index date was defined as the date of death for cases and copied for matched controls. For the present study, controls younger than the youngest suicide decedent (10 years) were excluded, leaving 297,034 total controls.

### 1.2 Measures

All MHRN‐affiliated health systems maintain a Virtual Data Warehouse (VDW) that houses electronic medical records and insurance claims data for enrolled health plan members in a standardized format. For the current study, the VDW was used to identify all health system encounters for cases and controls in the year preceding the index date. Suicide deaths were identified from official government mortality records using ICD‐10 codes V60‐X84 and Y87.0 matched with the VDW. Cancer diagnoses were extracted based on ICD‐9 codes 140–209 and were grouped according to tumor site as follows: head and neck (140–149, 160–161), gastrointestinal (150–159, 176.3, 209), lung (162–165), musculoskeletal (170–171, 176.1–0.176.2, 176.5–176.9), melanoma (172), nonmelanoma skin (173, 176.0), breast (174–175), gynecological (179–184), genitourinary (185–189), central nervous system (190–192), endocrine (193–195), lymph/blood (200–208), and metastatic (196–199). Psychiatric diagnoses were extracted based on ICD‐9 codes 290–319 and were grouped according to type as follows: alcohol use disorder (291, 303, 305.0), drug use disorder (292, 304, 305.2–305.9), psychotic disorders (291.1, 292.1, 293.8, 295, 297, 298) analyzed in aggregate and separating out schizophrenic spectrum (295) and nonschizophrenic disorders (297, 298), affective disorders (292.84, 293.83, 296), bipolar disorder (296.0, 296.1, 296.4–296.9301.1), depressive disorders (296.2, 296.3, 296.82, 300.4, 301.1309, 311), anxiety disorders (293.84, 300.0–300.3, 308, 309.2, 309.81), posttraumatic stress disorder (309.81), dementia (290, 291.2, 292.82, 294), eating disorders (307.1, 307.5), personality disorders (300.4, 301), gender dysphoria (302.6, 302.85), autism spectrum disorder (299), pediatric disorders (309.2, 312, 313, 314) analyzed in aggregate and separating out attention deficit disorder (314) and conduct disorder (312, 313.81, 314.2), and, other disorders (290–319 not otherwise specified, except drug use disorders in remission and tobacco use disorder).

Demographic information on age and sex was also available from the VDW; however, race/ethnicity was not available from several sites prior to 2008 and so was not considered in the analysis. Type of insurance (Commercial, Medicare, Medicaid, or Private Pay) was also included. For each person, median neighborhood income was derived from the patient's address and Census block data and recorded as a continuous variable in $10,000 increments.

### 1.3 Analysis

Due to the number of cancer and mental health subtypes being considered, an iterative model‐building approach was taken to strike a balance between model flexibility and parsimony. In the first step, conditional logistic regression models were used to calculate the age‐ and sex‐adjusted odds of suicide for each subtype of cancer and each mental disorder described above. Age and sex were considered nuisance parameters; sex was adjusted for using a binary indicator, and age was adjusted for using a natural spline with seven knots. All models were conditional on the MHRN site. Individual odds ratios are reported for all cancer disease sites and mental health diagnoses.

In the next step, cancer and mental health diagnoses were pooled to create more parsimonious models. Because cancer disease site, as indicated by the ICD‐9 code, was the only information available in the VDW by which to estimate prognosis, a three‐level cancer variable was created based on the average 5‐year survival rate for each cancer disease site without respect to the stage of disease.^16^ Cancer disease sites with a 5‐year survival rate ≥68% (the average survival rate for all cancer) were considered “good prognosis,” cancers with a survival rate of 34–68% were considered “moderate prognosis,” and cancers with a survival rate of ≤33% were considered “poor prognosis.” Cutoffs were arbitrary, intended to approximate prognosis while maintaining a sufficient number of cases in each bin to maximize power. Cancer stage, histology, and other tumor characteristics were not available for analysis. The BIC for a model incorporating this pooled variable was compared against models which included a binary indicator for any cancer and indicators for each separate cancer site. We elected to take a data‐driven approach to pool psychiatric diagnoses, and so a three‐level psychiatric variable was created by pooling individual diagnoses with similar adjusted odds ratios for suicide and dropping those with a nonsignificant association after multiple comparison adjustments. Patients with multiple diagnoses were assigned to the most severe category.

The final conditional logistic regression model was fitted, including age and sex, the pooled cancer and mental health diagnosis variables and interaction between them, and patient insurance type and median neighborhood income. All analyses were conducted using R version 4.1.2.

## 2 RESULTS

Sample characteristics, stratified by study class (cases vs controls), are shown in Table 1. Overall, cases were more likely to be male, older, insured by Medicare as opposed to commercial insurance, and live in neighborhoods with slightly lower median household incomes.

**TABLE 1:** Demographic characteristics of the study sample

	Controls, % (*n*) or mean (SD) (*n* = 297,034)	Cases, % (*n*) or mean (SD) (*n* = 3330)	*p* value
Male	46.8 (139,076)	77.2 (2571)	<0.001
Age (years)	43.8 (19.8)	50.0 (19.2)	<0.001
Commercial insurance	74.0 (219,663)	63.7 (2120)	<0.001
Private pay	3.4 (10,186)	4.0 (132)	
Medicare	13.7 (40,608)	23.0 (765)	
Medicaid	2.0 (5958)	2.2 (74)	
Other/unknown insurance	6.9 (20,619)	7.2 (239)	
Median neighborhood income	68.0 (28.7)	65.9 (28.2)	<0.001

Adjusting for age and sex, a past‐year cancer diagnosis was associated with a 78% increase in the relative odds of suicide (AOR 1.78, 95% CI 1.57–2.02). Relative odds of suicide by a specific cancer site are shown in Table 2. The BIC for a model including only a binary indicator for any cancer dx was 68,244; the BIC for a model incorporating each cancer site separately was 68,259; the BIC for a model including the pooled “good/moderate/poor prognosis” cancer variable was 68,158, indicating that pooling cancer diagnoses by expected mortality rate fit the data best. Good prognosis cancers were not associated with a significant increase in suicide risk; moderate prognosis cancers were associated with a 56% increase in relative risk (AOR 1.56, 1.16–2.11); poor prognosis cancers were associated with an almost five‐fold increase in suicide risk (AOR 4.76, 3.94–5.75).

**TABLE 2:** Odds of suicide among individuals with a cancer diagnosis in the past 1 year by cancer disease site

Cancer disease site	Control, % (*n*) (*n* = 297,034)	Case, % (*n*) (*n* = 3330)	AOR (95% CI)*	*p* value
Any cancer	3.9 (11,474)	8.6 (287)	1.78 (1.57–2.02)	<0.001
Head and neck	0.1 (262)	0.5 (18)	3.88 (2.44–6.17)	<0.001
Gastrointestinal	0.3 (994)	1.4 (48)	3.07 (2.30–4.09)	<0.001
Lung	0.2 (497)	1.1 (37)	4.98 (3.59–6.91)	<0.001
Musculoskeletal	0.1 (168)	0.1 (<10)	**	**
Melanoma	0.2 (452)	0.2 (<10)	**	**
Nonmelanoma skin	1.0 (3086)	1.9 (63)	1.23 (0.96–1.59)	0.104
Breast	0.8 (2309)	0.6 (21)	1.25 (0.81–1.93)	0.308
Gynecological	0.2 (535)	0.1 (<10)	**	**
Genitourinary	0.9 (2525)	2.7 (91)	1.59 (1.28–1.97)	<0.001
Central nervous system	0.1 (225)	0.2 (<10)	**	**
Endocrine	0.2 (470)	0.3 (10)	1.76 (0.94–3.27)	0.075
Lymph and blood	0.4 (1066)	1.1 (36)	2.15 (1.55–2.99)	<0.001
Metastatic	0.4 (1215)	2.7 (89)	5.42 (4.38–6.70)	<0.001
“Good prognosis” cancer	2.6 (7660)	3.8 (127)	1.16 (0.97–1.39)	0.109
“Mod. prognosis” cancer	0.7 (1986)	1.3 (44)	1.56 (1.16–2.11)	0.003
“Poor prognosis” cancer	0.6 (1828)	3.5 (116)	4.76 (3.94–5.75)	<0.001

*Note*: Good prognosis: lip, skin, genitourinary, eye, thyroid, and Hodgkin's disease; moderate prognosis: all types not listed above and below; poor prognosis: esophagus, stomach, liver, pancreas, lung, brain, and CNS, metastatic at any site (indicated by ICD‐9 codes 196–199).

* Logistic regression models conditional on site and controlling for sex and age.

** Categories with fewer than 10 cases were not analyzed separately though were included in the pooled prognosis variable.

Adjusting for age and sex, a past‐year diagnosis of any psychiatric disorder was associated with greater than a six‐fold increase in the relative risk of suicide (AOR 6.76, 6.31–7.25). Relative odds of suicide by specific disorder class are shown in Table 3. Dementia was not significantly associated with suicide. Attention deficit and pediatric disorder excepting conduct disorder had the weakest associations, and when pooled were not associated with suicide when controlling for comorbid conditions. Anxiety, eating, conduct, depression, personality, and other unspecified disorders had the next strongest associations, and when pooled increased suicide risk over three‐fold (AOR 3.40, 3.07–3.77). Posttraumatic stress, nonbipolar affective (e.g., unspecified mood disorder due to known physiological condition), and nonschizophrenic psychotic disorders were more strongly associated, and when pooled increased suicide risk over eight‐fold (AOR 8.33, 7.55–9.18). Finally, alcohol and drug use, schizophrenia spectrum, and bipolar disorders had the strongest associations, and when pooled increased suicide risk over 16‐fold (AOR 16.38, 15.01–127.88). In a post hoc analysis, we assessed whether the results differed depending on the order of cancer and psychiatric diagnoses. Results were similar to the unordered analysis, although small sample sizes for many diagnoses likely limited statistical power.

**TABLE 3:** Odds of suicide among individuals with a mental health diagnosis in the past 1 year

Diagnosis	Control, % (*n*) (*n* = 297,034)	Case, % (*n*) (*n* = 3330)	AOR (95% CI)*	*p* value
Any mental health	17.5 (52,074)	56.9 (1896)	6.76 (6.31–7.25)	<0.001
Affective/mood disorder	5.3 (15,608)	32.2 (1071)	9.58 (8.90–10.32)	<0.001
Alcohol use disorder	1.1 (3157)	13.2 (440)	10.68 (9.65–11.82)	<0.001
Attention deficit disorder	1.8 (5199)	3.0 (101)	2.35 (1.92–2.87)	<0.001
Anxiety/adjustment disorder	7.4 (21,956)	28.5 (949)	5.78 (5.36–6.24)	<0.001
Autism spectrum disorder	0.2 (459)	0.2 (<10)	**	**
Bipolar/manic disorder	0.8 (2239)	7.6 (254)	11.32 (9.95–12.87)	<0.001
Conduct disorder	0.3 (884)	1.3 (42)	6.89 (5.05–9.39)	<0.001
Dementia	0.5 (1462)	0.9 (31)	1.08 (0.75–1.55)	0.683
Depressive disorder	9.6 (28,415)	41.4 (1380)	7.65 (7.13–8.20)	<0.001
Drug use disorder	0.8 (2399)	9.0 (299)	10.70 (9.49–12.06)	<0.001
Eating disorder	0.2 (540)	0.7 (23)	6.51 (4.32–9.83)	<0.001
Gender dysphoria	<0.01 (24)	0.1 (<10)	**	**
Other psychotic disorder	0.4 (1127)	4.3 (143)	8.82 (7.43–10.46)	<0.001
Pediatric disorders	2.0 (5878)	4.1 (137)	2.90 (2.43–3.45)	<0.001
Personality disorder	1.6 (4871)	10.4 (346)	7.60 (6.79–8.50)	<0.001
Psychotic disorders	0.5 (1558)	5.5 (183)	8.47 (7.28–9.85)	<0.001
Posttraumatic stress disorder	0.5 (1548)	3.6 (120)	8.87 (7.39–10.65)	<0.001
Schizophrenic spectrum disorder	0.2 (494)	2.4 (79)	12.00 (9.60–15.03)	<0.001
Other disorders	3.3 (9855)	14.9 (497)	4.79 (4.35–5.28)	<0.001
“Low risk” disorder	0.9 (2527)	0.4 (12)	1.05 (0.59–1.85)	0.877
“Med‐low risk” disorder	9.6 (28,571)	15.4 (514)	3.40 (3.07–3.77)	<0.001
“Med‐high‐risk” disorder	4.5 (13,378)	17.4 (581)	8.33 (7.55–9.18)	<0.001
“High‐risk” disorder	2.4 (7138)	23.5 (782)	16.38 (15.01–17.88	<0.001

*Note*: Low risk: autism spectrum, attention deficit, and other pediatric disorders; med‐low risk: anxiety, conduct, depressive, eating, personality, and other (unspecified) disorders; med‐high risk: affective, posttraumatic stress, and nonschizophrenic psychotic disorders; high risk: alcohol use, drug use, bipolar/manic, schizophrenic spectrum disorders, and gender dysphoria.

* Logistic regression models conditional on site and controlling for sex and age.

** Categories with fewer than 10 cases are not presented separately though were included in the pooled “low/med/high” risk variable.

**FIGURE 1:**
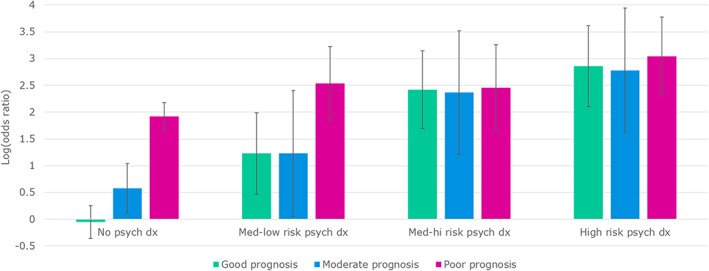
Suicide risk by cancer diagnosis, stratified by concurrent psychiatric diagnosis

Both psychiatric and cancer diagnoses remained significantly associated with suicide when controlling for each other. Finally, multivariate model coefficients are shown in Table 4. There was a significant, negative statistical interaction between “poor prognosis” cancer diagnosis and all classes of psychiatric disorders, such that concurrent diagnoses of a poor prognosis cancer and “high” or “medium‐high risk” psychiatric disorders did not appear to increase the relative risk of suicide compared with psychiatric diagnoses alone; for patients with concurrent poor prognosis cancer and “medium‐low risk” psychiatric diagnoses, the additional risk was attenuated but not as strongly as for higher risk psychiatric disorders. A similar trend was observed for “moderate prognosis” cancer diagnoses, but this interaction was not statistically significant. See Figure 1 for an illustration of how the observed suicide risk changed with concurrent diagnoses.

**TABLE 4:** Log‐odds of suicide in multivariate conditional logistic regression

Variable^1^	Coefficient	SE	*p* value
“Good prognosis” cancer dx	−0.007	0.158	0.965
“Moderate prognosis” cancer dx	0.581	0.232	0.012
“Poor prognosis” cancer dx	1.923	0.136	<0.001
“Medium‐low risk” MH dx	1.122	0.056	<0.001
“Medium‐high risk” MH dx	2.136	0.053	<0.001
“High‐risk” MH dx	2.811	0.047	<0.001
Good prognosis cancer dx * Med‐low risk MH dx	0.116	0.259	0.653
Mod. prognosis cancer dx * Med‐low risk MH dx	−0.478	0.446	0.283
Poor prognosis cancer dx * Med‐low risk MH dx	−0.503	0.242	0.038
Good prognosis cancer dx * Med–high‐risk MH dx	0.294	0.237	0.214
Mod. prognosis cancer dx * Med–high‐risk MH dx	−0.343	0.408	0.401
Poor prognosis cancer dx * Med–high‐risk MH dx	−1.603	0.333	<0.001
Good prognosis cancer dx * High‐risk MH dx	0.059	0.260	0.822
Mod. prognosis cancer dx * High‐risk MH dx	−0.611	0.424	0.150
Poor prognosis cancer dx * High‐risk MH dx	−1.690	0.280	<0.001
Household income^2^ (per $10,000)	−0.017	0.007	0.010
No insurance (private pay)^3^	0.296	0.092	0.001
Medicare	0.476	0.079	<0.001
Medicaid	0.185	0.124	0.135
Unknown insurance type	0.684	0.200	0.001

^1^ Sex and age were treated as nuisance parameters and are not shown for simplicity, since natural spline coefficients are especially difficult to interpret.

^2^ Household income and insurance did not have interactive effects with a cancer diagnosis but were included in the model due to significant main effects.

^3^ Commercial insurance is used as a reference group.

## 3 DISCUSSION

This study expanded on prior work in the US healthcare system by examining statistical interaction between concurrent diagnoses of cancer and a psychiatric disorder on risk for suicide, and parsing both diagnoses into more specific groups based on cancer site, as a marker for disease prognosis, and type of psychiatric disorder. Importantly, we did not find evidence for a synergistic effect between concurrent cancer and psychiatric diagnoses. Both diagnoses are associated with independent increases in suicide risk, but concurrent diagnoses do not appear to increase risk beyond what would be expected from a simple multiplicative relationship. In fact, we found the opposite for most psychiatric patients: for persons diagnosed with moderate to severe psychiatric disorders a concurrent cancer diagnosis did not increase their risk of suicide significantly compared to persons with those psychiatric disorders and no cancer. These patients are still at the highest risk for suicide, but cancer diagnoses do not appear to increase this risk further. This may be because these patients are more likely to receive intensive mental health interventions for their psychiatric disorder, which then mitigate the impact of other diagnoses. Or, if we consider a diathesis‐stress model for suicide risk, there may be an upper limit to how much stress contributes to suicide risk and severe psychiatric disorders are sufficient in and of themselves to hit that threshold.

We found that diagnoses of cancer in sites with a high average 5‐year survival rate did not significantly increase the risk of suicide for any patients, while cancers in sites with the lowest survival rates increased risk significantly more than cancers in other sites. Other studies have reported similar findings,^4^ and it is well established that a worse prognosis, usually measured either by late‐stage disease or cancer in a site with high average mortality, is associated with greater suicide risk.^17^, ^18^, ^19^, ^20^ For example, a Swedish registry study of men with prostate cancer found that suicide risk was not significantly elevated among men with PSA‐detected T1c tumors (less severe disease) but was significantly higher for men with locally advanced and metastatic tumors.^21^ The authors note that it is important for healthcare providers to look beyond a simple diagnosis when assessing which patients are most likely to benefit from additional suicide risk screening.^22^ Studies have also identified other characteristics that bear considering. Pain and lower levels of physical functioning have been reported to be associated with suicidal ideation in cancer patients in multiple studies.^23^ In the current study, we were unable to evaluate other characteristics that would affect the patient's prognosis. However, considering the available literature, it seems most likely that a prognosis that takes the complete clinical picture into account, rather than just the primary cancer site, should guide physician decision‐making around suicide risk screening and intervention for recently diagnosed cancer patients.

Several studies have followed cancer patients up to 25 years after initial diagnosis and assessed suicide risk over that time, and they generally find that risk is highest immediately following diagnosis (1–3 months) but remains elevated compared to the general population for up to 1–4 years.^17^, ^18^, ^19^, ^20^ The reasons for the increased risk are not understood but elucidating them is an important task that will help guide preventive efforts. There is currently no gold standard for suicide prevention among cancer patients, however, various pharmacotherapies, psychosocial interventions, and physical exercise have all shown some efficacy at reducing factors presumed to be associated with suicide risk (symptoms of anxiety and depression, pain and fatigue, and general quality of life measures).^24^

The current study also found that diagnoses with all psychiatric disorders except for dementia were associated with increased suicide risk, although most pediatric disorders were not significantly associated when controlling for other, comorbid psychiatric disorders. Prior research has confirmed that most psychiatric disorders are associated with suicidal thoughts and behaviors,^5^ and emerging literature suggests that a latent “general psychopathology factor” is more important for predicting suicide risk than any specific disorder.^8^ The extent to which differences in the relative risk of suicide between different psychiatric disorders observed in our study represent merely differences in the average level of this general psychopathology factor between disorders versus truly disorder‐specific risk mechanisms could not be assessed but is an area that deserves further research.

This study has limitations to consider. The original case–control study was designed to identify previously unrecognized, near‐term risk factors for suicide death that are documented in healthcare settings. We expanded on this aim to examine how two of these factors, cancer and psychiatric diagnoses, interact with each other. However, the study was not designed to assess either mediation or moderation between risk factors. Doing so would require knowing when the first diagnoses were made (i.e., the temporal ordering of cancer and psychiatric diagnoses) and in a retrospective study looking back only 1 year from the index date, this information is not available. Lack of information on diagnoses made prior to 1 year from the index date may contribute to the post hoc observation that the results did not change significantly when considering which diagnosis, cancer or psychiatric disorder, appeared first in our limited data set. Competing risks (i.e., people with aggressive cancer at high risk for suicide might die of the disease before completing suicide) may cause an underestimation of the risk due to cancer with a poor prognosis. We also relied on health system records, and some diagnoses may not have been recorded even if patients had symptoms. Similarly, some psychiatric disorders are chronic, whereas others are episodic, with symptoms that appear and disappear over time. A diagnosis, therefore, does not necessarily confirm what symptoms were present at the time of suicide among cases. Details about cancer stage and histology were not available, only ICD‐9 codes, and so disease site was used as a rough approximation of prognosis. As prognosis within each cancer site varies, this grouping is imperfect and may obscure an interaction between psychiatric diagnosis and cancer diagnosis. Although the ICD‐9 does include codes for metastasized cancer (196–199) which may be inferred to represent severe disease and which we made full use of, other than these codes we have no way to distinguish patient prognosis over the study duration. Neither did we know where patients were on their cancer journey; ICD‐9 codes could represent new diagnoses, recurrence, or prevalent diagnoses for patients in treatment, and we had no way to disentangle these potentially different patient groups. Other characteristics beyond prognosis that were not measured in the current study may be important factors in determining suicide risk, including social support,^25^ pain,^26^ or sequelae associated with the treatment of particular cancers (e.g., loss of fertility, disfiguring scars). Finally, although we included a geographically diverse group of US healthcare systems, all participating systems are well resourced and patients almost all had some form of insurance. Results may not be generalizable to smaller healthcare systems and/or to patients without insurance.

Despite these limitations, this study is one of the first to examine the interaction between cancer and psychiatric disorders on suicide risk. By pooling data from nine different healthcare systems across the US, we were able to obtain a sufficient sample size to examine specific cancer and psychiatric diagnoses and assess their association with the (fortunately) rare outcome of suicide mortality. We found that moderate‐and poor‐prognosis cancer diagnoses increased the risk of suicide among patients with no or mild psychiatric diagnoses. However, patients with moderate to severe psychiatric diagnoses remain the patients at the highest risk of suicide, and suicide risk is not increased among these patients with a concurrent cancer diagnosis. These results point to the need for widespread suicide prevention efforts in healthcare systems. Research into the mechanism(s) by which cancer diagnoses increase suicide risk can help further improve the targeting of preventive efforts and may also suggest more specific targets for intervention, for example, alleviating feelings of hopelessness, chronic pain, or financial stress, any or all of which might theoretically mediate the robust association between cancer and suicide.

## ETHICS STATEMENT

IRB approval (expedited) was granted from the individual IRBs at each participating institution. Because the study involved only de‐identified, routinely collected information from the medical record, patient informed consent was deemed not necessary.

## AUTHOR CONTRIBUTIONS

Conceptualization – Drs. Kahn, Tam, and Ahmedani.

Data Curation – Dr. Westphal.

Formal Analysis – Dr. Kahn.

Funding Acquisition – Drs. Simon, Ahmedani.

Investigation – Drs. Simon, Owen‐Smith, Rossom, Beck, Lynch, Daida, Lu, Waring, Frank, and Akinyemi.

Methodology – Dr. Kahn.

Writing, original draft – Drs. Kahn, Tam, Felton, and Ahmedani.

## FUNDING INFORMATION

This work was funded by the National Institute of Mental Health (grant no. T32MH12579‐01).

## CONFLICT OF INTEREST

None.

## Data Availability

As data were derived from individual medical records, research data are not shared.
